# Medical College Fees

**Published:** 1868-10-01

**Authors:** 


### *EDITORIAL.*

---

### *Medical College Fees—Free Medical Education.*

A cheap advertising medium of a cheap school in Cincinnati, perpetrates the following, which we place in parallel columns for the edification of our readers. We shall not give the name of the medium, as it is sufficiently indicated in a recent number of the *Leavenworth Herald.* We did not before understand the concentrated scorn involved in the allusion by our Leavenworth *confrere*, to the "*Cincinnati College of Medicine and Surgery:*

THE MEDIUM, AUGUST, 1868.

We maintain, and we believe we will be sustained in our views by a right-thinking community, that the true method to elevate the profession is to do away as far as possible with pecuniary obstacles in entering the profession, and to place at a high standard the attainments necessary for graduation. Want of wealth, then, would not prevent the poor man, eminently qualified, for competing for the honors of the profession, while the high qualifications required for graduation would deter both rich and poor alike, unqualified, from seeking after them.

THE MEDIUM, OCTOBER, 1868.

FREE MEDICAL EDUCATION.—The editor of the *Chicago Medical Journal*, in its column of editorials, which are served up in such a novel and interesting manner, says that the *Medical Repertory* is in favor of free medical education—the instructors receiving no compensation. When we recollect that he belongs to Chicago, we are not surprised at the statement. People up there have a loose way of stating and doing things, and the editor undoubtedly believes in free lying if he doesn't in free medical education.

When in connection with this it is known that the "college" this medium represents, puts its professors' tickets at *twenty dollars* "for the lot," we do not wonder at our belief in the "free lying" of the editor of the *Medium*, who, we believe, is a "Professor" in that delectable concern. "High qualifications" (at least of a moral sort) will certainly prove no hindrance to graduation at the "Cincinnati College of Medicine and Surgery." We turn this creature over to Brothers Logan and Sinks.

